# Introducing an Expanded Value Framework in Health Technology Assessment of Vaccines

**DOI:** 10.3390/jmahp14020024

**Published:** 2026-04-28

**Authors:** Farzaneh Eslami, Thi Hao Pham, Angga P. Kautsar, Cao Ba Khuong, Cornelis Boersma, Mondher Toumi, Jurjen van der Schans, Maarten J. Postma

**Affiliations:** 1 Unit of Global Health, Department of Health Sciences, University Medical Center Groningen (UMCG), University of Groningen, 9713 GZ Groningen, The Netherlands; t.h.pham@umcg.nl (T.H.P.); a.p.kautsar@rug.nl (A.P.K.); c.ba.khuong@umcg.nl (C.B.K.); cornelisboersma@health-ecore.com (C.B.); j.van.der.schans@rug.nl (J.v.d.S.); m.j.postma@rug.nl (M.J.P.); 2 Department of Pharmaceutics and Pharmaceutical Technology, Faculty of Pharmacy, Universitas Padjadjaran, Sumedang 45363, Indonesia; 3 Center of Excellence for Pharmaceutical Care Innovation, Universitas Padjadjaran, Bandung 45363, Indonesia; 4 Faculty of Public Health, Thai Nguyen University of Medicine and Pharmacy, Thai Nguyen 250000, Vietnam; 5 Health-Ecore, 3704 HE Zeist, The Netherlands; 6 Health-Ecore, 9728 JT Groningen, The Netherlands; 7 Department of Management Sciences, Open University, 6419 AT Heerlen, The Netherlands; 8 Health Services Research and Quality of Life Center, Aix-Marseille University, 13385 Marseille, France; mto@inovintell.com; 9 InovIntell, 30-605 Krakow, Poland; 10 Department of Economics, Econometrics and Finance, Faculty of Economics & Business, University of Groningen, 9747 AJ Groningen, The Netherlands; 11 Division of Pharmacology & Therapy, Faculty of Medicine, Universitas Airlangga, Surabaya 60115, Indonesia

**Keywords:** vaccination, vaccines, health technology assessment, cost-effectiveness, value framework, economic evaluation

## Abstract

Health Technology Assessment (HTA) frameworks increasingly recognize the broader value elements of vaccines; however, their adoption remains inconsistent across jurisdictions and often incomplete in practice. Many HTA processes continue to prioritize narrow clinical outcomes and direct costs, leading to the underrepresentation of the full preventive and long-term benefits of vaccination. Building on the ISPOR “Elements of Value” framework and recent evidence, this study adapts and expands existing models specifically for vaccines to enhance HTA applicability in both high-income and resource-limited settings. We introduce an updated vaccine value framework comprising 21 distinct value elements. Notably, the original model was expanded by introducing four entirely new value drivers: (1) real-world evidence; (2) control of antimicrobial resistance; (3) health system strengthening; and (4) environmental impact. Additionally, existing elements were refined, such as broadening “fear of contagion” to “peace of mind” and expanding “productivity” to capture education and leisure gains. We map these elements to potential data sources and methodological tools to facilitate their inclusion in HTA. This study offers an operational, holistic, and context-sensitive framework that reflects current advancements in assessment. By capturing the full spectrum of vaccine value, this framework aims to support more comprehensive, transparent, and equitable HTA decision-making for global immunization programs, while considering conceptual overlap between value elements to reduce the risk of double counting.

## 1. Introduction

Health Technology Assessment (HTA) typically evaluates interventions based on clinical effectiveness, cost-effectiveness (C-E), medical need, and patient-centered outcomes such as morbidity, mortality, and quality of life (QoL) [1,2]. While HTA systems differ across countries, with countries such as the UK, Sweden, and the Netherlands emphasizing C-E, others like France and Germany prioritize clinical criteria [3,4]. Traditional models were originally designed for therapeutic drugs, mainly focusing on direct outcomes and healthcare costs [5]. Recent initiatives such as the BRAVE framework have attempted to systematically identify and prioritize broader value elements of vaccination that are not consistently included in current HTA practices [4]. Vaccines are often undervalued in HTA because conventional approaches incompletely capture their broader societal and economic benefits, particularly in low- and middle-income countries (LMICs) [6].

Vaccines, however, generate broader benefits, including herd protection, reduced antimicrobial resistance (AMR), and productivity gains, which extend beyond these narrow metrics [7,8,9]. Several economic evaluations and HTA analyses of vaccination programs have been conducted, although many focus primarily on traditional cost-effectiveness outcomes [10]. While some countries have begun to incorporate such indirect effects, their inclusion remains inconsistent and often limited in practice [11,12,13].

High-income countries (HICs) generally have stronger HTA capacity, better data systems, and more mature decision-making processes, enabling broader valuation of vaccines [14,15]. Yet even in HICs, several important benefits of vaccines remain insufficiently or inconsistently explored [15]. In contrast, many LMICs focus primarily on efficacy and C-E, constrained by limited resources, data gaps, and lower institutional awareness of HTA’s potential [16,17]. As a result, value elements such as macroeconomic gains, social equity, and health system strengthening are often omitted, contributing to inconsistent vaccine prioritization and access across settings [12,18].

The ISPOR framework, also known as the value flower [19], conceptualizes the diverse impacts of medical technologies. The fact that several petals remain abstract indicates the poor operationalization of some vaccines [20,21]. Recent discussions on vaccine assessment have also emphasized the need to better integrate public health perspectives and advisory bodies, such as National Immunization Technical Advisory Groups (NITAGs), with HTA processes to ensure that broader vaccine value dimensions are adequately considered [22]. Therefore, this paper aims to adapt and refine the ISPOR value framework specifically for vaccination, improving measurability and applicability to support more consistent, holistic vaccine evaluations across health systems, including LMICs.

## 2. Materials and Methods

To inform the development of the expanded vaccine value framework, a targeted rapid scoping search of the literature was conducted. The aim of this search was not to perform a systematic review but to identify conceptual and methodological publications discussing value frameworks, broader economic value, and HTA approaches relevant to vaccination.

Searches were conducted in PubMed and Scopus using combinations of keywords related to vaccination, value frameworks, economic value, and HTA (e.g., “vaccine”, “vaccination”, “value framework”, “economic value”, “societal value”, “health technology assessment”, and “economic evaluation”). Searches were restricted to title and abstract fields in PubMed and to title, abstract, and keyword fields in Scopus, focusing on publications explicitly addressing vaccine value frameworks or broader value concepts relevant to HTA. The search covered the literature published between 2010 and 2025, with 2010 chosen as the lower bound to capture relatively recent developments in vaccine value frameworks and HTA. Because the aim of this search was to inform conceptual framework development rather than to conduct a comprehensive systematic evidence synthesis, the search strategy was designed to identify key conceptual and methodological publications rather than exhaustively capture all clinical studies.

The search identified 189 records (PubMed: 105; Scopus: 84). After removal of duplicates, 148 records remained for title and abstract screening. Publications were included if they discussed conceptual frameworks for HTA, broader value elements of vaccination, or methodological approaches for incorporating societal and economic value into vaccine evaluation. Studies focusing exclusively on clinical efficacy, epidemiology, or immunology without discussion of value assessment were excluded.

Following title and abstract screening, 33 publications were assessed in full text for relevance. Of these, 29 key publications were included to inform the development of the framework. Additional relevant publications were identified through screening of the references for key articles.

The identified literature was synthesized using a best-fit framework synthesis approach, starting from the ISPOR “Elements of Value” framework proposed by Lakdawalla et al. [19]. Value elements identified in the literature were compared with the original ISPOR framework and mapped onto existing petals. Value elements were consistently represented when they appeared across multiple conceptual or methodological publications assessing vaccine value. When vaccine-specific value dimensions repeatedly appeared in the literature but were not explicitly represented in the ISPOR framework, they were incorporated as additional elements and extensions of the framework. This process resulted in the identification of 21 value elements relevant to vaccine evaluation, which are described and operationalized in the proposed framework. Where direct quantitative evidence is limited, proxy indicators (e.g., labor market statistics on the impacts of productivity) or qualitative evidence (e.g., policy analyses describing resilience of health systems) may be used to inform the assessment of specific value elements. The full search strings used for PubMed and Scopus are provided in Supplementary Materials File S1.

## 3. Results

**Figure 1:**
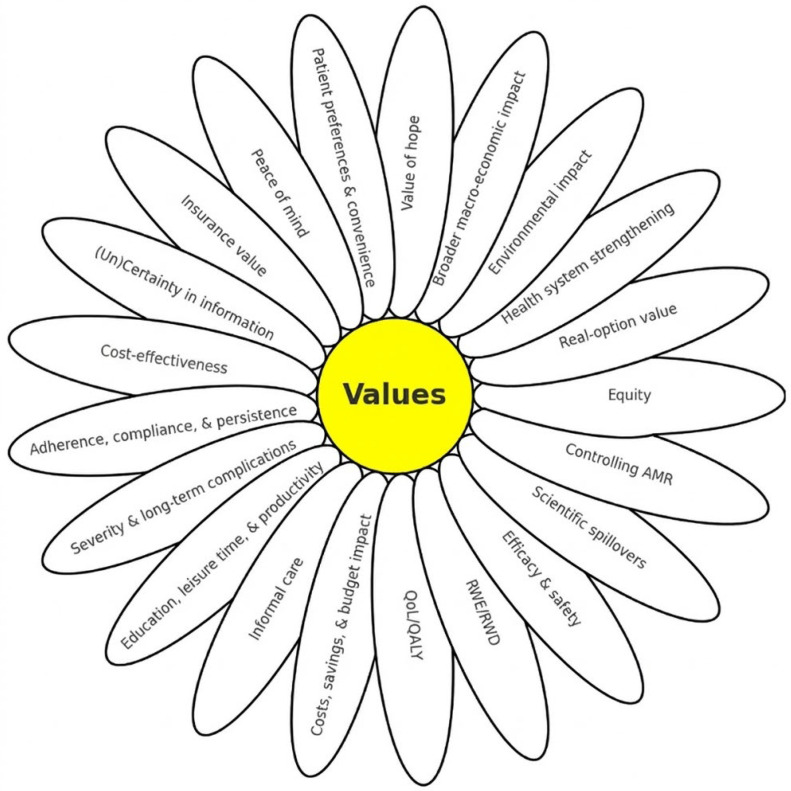
Revised value framework for vaccine assessment. AMR: Antimicrobial Resistance; RWE/RWD: Real World Evidence/Real World Data; QALY: Quality-Adjusted Life Years; QoL: Quality of Life.

The number of value elements was not predefined but emerged from the framework synthesis process. Starting from the original ISPOR value framework, vaccine-specific dimensions inadequately reflected by the existing petals were introduced as additional elements, resulting in a total of 21 value elements. Figure 1 displays the extended value framework, while Table 1 provides illustrative examples linking each value element to corresponding HTA metrics and typical data sources. The table is not intended to be exhaustive, as multiple analytical tools may be appropriate depending on the HTA context and data availability. Instead, it serves as an operational guide for integrating broader value dimensions in vaccine HTAs, including the context of LMICs. To avoid repetitive descriptions across the individual value elements, Figure 1 and Table 1 summarize the overall framework structure, methodological tools, and data sources. The following sections, therefore, outline the conceptual relevance of each value element for vaccination.

### 3.1. Vaccine Efficacy and Safety

In the original ISPOR framework, efficacy and safety are not presented as separate value elements but are embedded within measures such as Quality-Adjusted Life Years (QALYs) gained [19]. In our vaccine-specific adaptation, these factors are treated as distinct components because they underpin preventive impact, public confidence, and regulatory decision-making.

A core value of vaccines lies in their demonstrated efficacy and safety, typically established through randomized controlled trials (RCTs). These trials assess a vaccine’s ability to prevent infection or disease (efficacy) and monitor for adverse effects (safety) under controlled conditions. Efficacy is often measured by immunological markers or reductions in confirmed cases, while safety is evaluated through short- and long-term monitoring of adverse events. These trial-based metrics form the basis for regulatory approval and HTAs [8], serving as essential inputs for cost-effectiveness analyses (CEAs), which are mandatory for vaccine recommendation and reimbursement decisions in many countries [23,24,25,26].

Data sources for this element are primarily derived from clinical trial reports and regulatory dossiers. QALYs and Disability-Adjusted Life Years (DALYs) are subsequently estimated from these outcomes through modeling.

### 3.2. Real World Evidence/Real World Data

In the original ISPOR value framework, real-world evidence (RWE) is not conceptualized as a standalone value element but rather as a data source or methodological input supporting other petals, such as QALYs, productivity, adherence, and uncertainty reduction [19]. In this framework, RWE is considered a distinct element because real-world vaccine performance strongly influences population-level impact and policy decisions, reflecting the increasing role of RWE in HTA. In this sense, RWE is not only a supporting data source but also a value-relevant dimension, as it enables the realization, validation, and interpretation of vaccine impact in real-world settings.

Real-world data (RWD) refers to routinely collected health information, while RWE represents the insights generated from analyzing these data [27,28,29]. RWE has been critical in recent vaccine evaluations; for example, UK surveillance systems use RWE to assess waning immunity and booster effectiveness during COVID-19 [27], while US RWD demonstrated herd protection following the introduction of PCV7 [28]. Such evidence captures effectiveness, safety, uptake, and healthcare use under real-life conditions; these are factors RCTs cannot observe over longer periods [30,31,32]. Within HTA, RWE strengthens external validity by informing adjusted C-E models and risk-based analyses, ensuring decisions reflect real-world vaccine performance [33].

Typical data sources include electronic health records (EHRs), registries, surveillance systems, and observational cohorts. These sources allow for adjusted CEA and risk modeling, providing a bridge between clinical trial efficacy and effective field performance.

### 3.3. Quality of Life

In the original ISPOR value framework, QoL is captured primarily through QALYs, which combine morbidity and mortality impacts into a single utility measure [19]. In this framework, QoL also reflects subjective aspects of health such as physical functioning, emotional well-being, and social participation that may not be fully captured by clinical endpoints alone.

In vaccination, the effects of QoL/QALYs arise from preventing symptomatic disease, reducing illness duration, and avoiding long-term complications not only in vaccinated individuals but also in indirectly protected populations such as parents or caregivers [34,35]. Long-term benefits are also seen when vaccines prevent chronic diseases or complications; for example, HPV and HBV vaccines reduce cancer risk and improve long-term QoL [7]. Composite indicators, such as burden-of-illness scores, help estimate vaccine-related improvements in health-related QoL [36]. Some HTAs also consider the population-level benefits of QoL, including herd protection and reduced transmission [9,37].

In HTA, QoL is typically included in QALY calculations using generic tools, such as the EuroQol 5-Dimension health questionnaire (EQ-5D) or Short Form-36 Health Survey (SF-36) and patient-reported outcomes (PROs) [38,39].

### 3.4. Cost, Savings, and Budget Impact

This element extends the “net costs” component of the original ISPOR framework (costs minus savings) by explicitly incorporating budget impact, reflecting its practical importance in HTA evaluations [19]. While Budget Impact Analysis (BIA) assesses the short-term financial implications for payers such as ministries of health, it remains closely linked to cost-effectiveness results, typically expressed through incremental cost-effectiveness ratios (ICERs) [40].

Vaccination programs entail direct costs, including procurement, storage, cold-chain logistics, delivery, and personnel, as well as downstream savings from reduced healthcare utilization, such as fewer hospitalizations, ICU admissions, and outpatient visits [37,41]. Indirect savings may also arise through herd protection. Overall, this value element reflects the balance between the upfront investment required for vaccination and the downstream cost savings and health benefits it generates; this information is essential for sustainable resource allocation in immunization programs [20]. The fact that the impact of budget can be considered relatively modest for vaccines can be interpreted as an additional value to net costs and QALYs.

Evidence for this element is commonly derived from cost-of-illness studies, healthcare utilization records, claims databases, and national reimbursement statistics, which inform both BIA and ICER calculations [29].

### 3.5. Informal Care

In the ISPOR value framework, informal caregiving is not presented as a standalone value element; instead, its impacts are potentially implicitly reflected through productivity losses and broader macroeconomic effects [19]. In this framework, informal care is considered a distinct element given its increasing importance in HTA guidelines, including those used in the Netherlands [7].

In vaccine evaluations, preventing the spread of infectious disease reduces the time families spend providing care, which is an effect not fully captured by traditional clinical outcomes focused on the patient alone [7,11]. Vaccination, therefore, generates value by limiting the amount of time spent caregiving, mitigating impacts on productivity, and reducing emotional burden, while also improving caregiver quality of life [7,11,25]. HTA systems adopting a societal perspective, such as in the Netherlands and Canada, increasingly incorporate informal care into economic evaluations to better capture the broader societal value of vaccination [7,12].

Informal care is typically valued through indirect cost or opportunity-cost approaches using data from time-use surveys, caregiver interviews, and labor market statistics [42].

### 3.6. Education, Leisure Time, and Productivity

“Productivity” is an established value element in the original ISPOR framework, capturing how illness affects individuals’ ability to work, often categorized as absenteeism and presenteeism [19]. In our adapted framework, this concept is expanded to include impacts on education and leisure time, recognizing that infectious diseases generate broader societal consequences beyond work loss [43,44].

In the vaccine context, preventing infection averts productivity losses and supports the continuity of education and social functioning. Loss of leisure time also represents a significant welfare loss, although careful consideration is needed to avoid double-counting with QALY reductions, as some utility measures may already implicitly capture these effects [45]. The COVID-19 pandemic highlighted the importance of considering a broader range of effects, as school closures and learning disruptions significantly influenced vaccine policy decisions regarding children and adolescents [46].

These effects are typically valued using human-capital or friction-cost methods based on labor statistics, time-use surveys, and educational attendance data [47,48,49]. When local data are unavailable, estimates from comparable countries may be used as proxies; for example, consistent German data on work absenteeism could be used to model impacts in the Netherlands, where consistent registration of such data is absent [50].

### 3.7. Severity and Long-Term Complications

The original ISPOR value framework identifies severity of disease as a distinct value element, reflecting societal preference to prioritize interventions for severe or life-threatening conditions [19]. In our updated framework, this element highlights the prevention of severe complications and long-term sequelae, such as those associated with meningococcal disease or chronic outcomes following viral infections [5].

In HTA, greater disease severity is often associated with higher willingness-to-pay (WTP) thresholds [19]; for example, in the Netherlands, acceptable costs per QALY increase from €20,000 for mild conditions to €80,000 for severe conditions [51]. Indeed, in a jurisdiction explicitly using severity as a criterion, as in the Netherlands, the lowest WTP threshold is not necessarily applied to vaccines anymore; instead, for more severe diseases, such as meningococcal B and influenza at older age, higher WTP thresholds are differentiated [52,53]. Within HTAs, severity is typically operationalized through severity-weighted QALYs, stratified cost-effectiveness thresholds, or analyses disaggregated by risk groups [54].

Common data sources include burden-of-disease metrics, hospital records, and national mortality statistics. Such data is integrated, for example, in the burden-of-disease indicator developed by Erasmus University (iMTA) [55], as applied in the Netherlands’ reimbursement system, which determines the appropriate threshold based on proportional shortfall.

### 3.8. Adherence, Compliance, and Persistence

In the original ISPOR value framework, this element was included to cover how consistent and correct use of a health technology affects its real-world performance [19]. In this framework, the concept is expanded to explicitly include adherence, compliance, and persistence, as these capture distinct aspects of vaccine use.

For vaccines, this concept translates to adherence or compliance to recommended schedules, completion of multi-dose series, and general uptake, all of which strongly influence population-level effectiveness. Unlike curative treatments, where adherence mainly affects individual outcomes, vaccine adherence has system-wide consequences, manifesting through coverage rates, which determine the degree of herd protection and overall impact of programs [56,57]. In CEAs, suboptimal adherence reduces the effective number of protected individuals, resulting in weaker health gains and diminished cost-effectiveness. By contrast, high adherence strengthens program value and supports more efficient resource allocation [58]. Furthermore, persistence as a concept can be considered to reflect potential persistent vaccination from one season to another for annual vaccinations, such as influenza. Considered in this way, persistence may generate benefits in the build-up of immunity and cross-protective effects from one seasonal vaccination to the next.

Common data sources include immunization registries, behavioral surveys, and routine coverage reports. In HTAs, these parameters are typically incorporated through scenario or sensitivity analyses that vary assumptions on uptake and completion rates [49].

### 3.9. Cost-Effectiveness

In the original ISPOR value framework, cost-effectiveness is not treated as a standalone value element but as an analytical approach that combines several petals, such as QALYs, costs, and productivity effects [19]. In this framework, cost-effectiveness is retained as a distinct domain because it synthesizes multiple upstream value elements into a decision-relevant metric widely used in HTA.

CEA integrates clinical and economic outcomes using QALYs, DALYs, and ICERs, supported by data on vaccine efficacy, safety, coverage, and disease severity [5,20,26]. In general, CEAs translate direct and indirect benefits, including reduced healthcare use, herd protection, and avoided long-term complications, into comparable units of value. This integrative metric identifies clear priorities and supports efficient allocation of scarce healthcare resources [59]. CEAs allow for the ranking of interventions and comparison with C-E thresholds when outcomes are expressed in QALYs. When QALYs are not available or irrelevant, CEAs expressed as the number of benefits gained for health can still provide informative decision support.

The required inputs include estimates of costs, savings, and health gains, which are combined to calculate ICERs or ICURs (Incremental Cost-Utility Ratio) and used to inform HTA decision-making.

### 3.10. (Un) Certainty in Information

In the original ISPOR value framework, a “reduction in uncertainty” reflects the value created when evidence becomes more reliable for decision-making [19]. In our framework, this element is crucial for vaccines, where uncertainty may arise from epidemiological assumptions, duration of protection, indirect effects, and implementation performance.

To reduce uncertainty, robust trial data and RWE are required, together with improved estimations of costs, savings, and health system capacity [60]. HTA agencies typically address uncertainty through (probabilistic) sensitivity analysis, value-of-information (VOI) analysis, and scenario testing [61,62]. Reducing uncertainty improves confidence in adopting and sustaining decisions, particularly for vaccines with long-term and indirect effects. In HTA, reducing uncertainty may also reflect the “value of knowing,” whereby improved evidence supports better-informed decisions for patients, clinicians, and policymakers [18]. In vaccines, uncertainty may arise from factors such as duration of protection, indirect effects, uptake, evolution of pathogens, and program implementation, highlighting the importance of post-implementation monitoring and continued generation of evidence [19].

Common data sources include epidemiological and surveillance data, RWE inputs, parameter distributions from the clinical and economic literature, and expert-elicited estimates [30]. Monitoring real-world vaccine performance, therefore, remains essential to reduce uncertainty about effectiveness, safety, and duration of protection, as highlighted during the COVID-19 pandemic [63].

### 3.11. Insurance Value

In the original ISPOR value framework, “insurance value” reflects the protection individuals gain from reduced uncertainty and financial risk when facing potential illness [19]. In this framework, this benefit is applied to vaccines, which act as curative medicines. Their benefit arises not only from preventing disease but also from protecting individuals from high and unexpected medical costs associated with severe infections [64].

This financial protection is reflected through approaches such as extended cost-effectiveness analysis (ECEA), which evaluates reductions in out-of-pocket spending and the risk of medical impoverishment. For example, ECEA, when applied to vaccination programs in Ethiopia, showed that vaccines can substantially reduce out-of-pocket health expenditures among low-income households, illustrating their contribution to financial risk protection and universal health coverage goals, particularly in low-resource settings where health inequities are often greater [65].

In HTA, insurance value can be incorporated using ECEA, willingness-to-pay (WTP) studies, or financial risk protection models supported by household-level data linking health outcomes and financial consequences [65,66].

### 3.12. Peace of Mind

In the original value framework, this element is described as “fear of contagion,” reflecting the psychological benefit of reduced anxiety about contracting infectious diseases [19]. Our framework incorporates the broader term peace of mind to capture the reassurance individuals experience from protection against infectious diseases through vaccination.

This value reflects the psychological and social benefits of feeling protected, which may increase vaccine uptake and trust in immunization programs [67]. At the community level, reduced transmission and protection of vulnerable groups may enhance collective reassurance and social and economic stability through herd protection; these benefits are often not captured in traditional cost-effectiveness metrics [7].

Although empirical implementation remains limited, peace of mind may be incorporated into HTAs that incorporate contingent valuation, WTP, or other stated-preference methods [67]. Common data sources include survey research, qualitative data, and stated-preference datasets.

### 3.13. Patient Preferences and Convenience

In the original value framework, patient preferences and convenience are not standalone value elements but can be considered as important determinants of other domains, particularly adherence [19]. In our framework, patient preferences are included as a separate petal because they are crucial in patient decision-making and influence uptake.

Convenience-related factors, such as travel distance, time required, administration route, schedule, and emotional discomfort or stigma, can influence willingness to start and complete vaccination [56]. For example, procedural inconvenience and embarrassment have been shown to reduce participation in cervical cancer screening, highlighting the importance of considering convenience in prevention strategies such as HPV vaccination [68]. In general, patient involvement in HTA is limited, necessitating enhanced measurement of these factors.

Patient preferences are commonly measured through discrete choice experiments and other stated-preference methods that evaluate trade-offs between attributes such as dosing schedules, injection type, and costs [69].

### 3.14. Value of Hope

In the original framework, the value of hope reflects the benefit patients derive from the possibility of better-than-expected outcomes, particularly in severe or life-threatening conditions where existing treatments offer limited improvement [19]. This framework includes patient preferences, risk tolerance, and the psychological benefit of maintaining optimism about future health [70].

This value is perhaps most relevant for therapeutic or next-generation vaccines, such as cancer immunotherapies, where vaccination could offer the possibility of returning to a healthier state [70,71]. For preventive vaccines, the value of hope may manifest more indirectly through reassurance among individuals facing elevated clinical or perceived risk [11,72]. For preventive vaccines, the value of hope manifests indirectly through reassurance among individuals facing elevated clinical or perceived risk. Therefore, in preventive vaccination, this element may be context-dependent, particularly in situations involving severe diseases or a high level of perceived risk. Although widely discussed, evidence supporting the systematic inclusion of hope in HTA remains limited, and its distinction from traditional QoL or survival benefits continues to be debated [71].

### 3.15. Broader Macroeconomic Impact

In the original ISPOR value framework, macroeconomic effects are not treated as a separate value element but are partly reflected through productivity and scientific spillovers [19]. In this framework, broader macroeconomic impact captures how vaccination can influence economic activity beyond the health sector.

Experience during the COVID-19 pandemic demonstrated that vaccination can reduce disruptions such as absence from work, business closures, declines in trade and tourism, and losses in gross domestic product (GDP) [43,73]. These effects extend beyond what traditional CEAs or BIAs typically capture. By preventing disease, vaccines help stabilize labor markets, reduce informal care needs, and support continuity of education and social functioning [43].

Macroeconomic impacts are commonly evaluated using computable general equilibrium (CGE) models or related macroeconomic approaches [43,73]. Typical data sources include GDP statistics, labor market data, and fiscal projections. Because these effects interact with elements such as productivity and informal care, macroeconomic impact represents a cross-cutting dimension that strengthens the broader societal value of vaccines, particularly in HTA frameworks adopting a societal perspective.

### 3.16. Environmental Impact

In the original ISPOR value framework, environmental effects were not included as a value element, reflecting the historically limited attention that HTAs have given to sustainability [19]. In our adapted framework, environmental impact is introduced as a new element, recognizing how vaccination programs generate resource use, waste, and greenhouse gas emissions across manufacturing, cold-chain storage, transport, and disposal.

Life-cycle assessment (LCA) methods are increasingly used to evaluate energy consumption, carbon emissions, and material waste in immunization supply chains [74]. Environmental considerations are becoming more relevant as health systems adopt climate-aligned policies and greener pharmaceutical procurement [75]. In vaccination programs, reducing dose requirements, minimizing cold-chain energy use, and lowering vaccine wastage can reduce both economic and environmental burdens [76].

This element can be incorporated into HTAs using LCA-based modeling supported by supply-chain audits, energy-use records, and emission registries [77]. Standardized measurement and consistent inclusion in HTAs may contribute to more sustainable and environmentally friendly immunization strategies.

### 3.17. Controlling Antimicrobial Resistance (AMR)

Controlling AMR represents an additional value element when evaluating vaccines [19,78]. A recent systematic review of 19 vaccine value frameworks showed that reductions in AMR are increasingly recognized as an important component of vaccines, even if not consistently operationalized in earlier frameworks [4,79]. Our framework, therefore, includes a reduction in AMR as a distinct element, reflecting its growing scientific and policy relevance.

Vaccines can mitigate AMR by preventing infections and reducing antibiotic use. For example, pneumococcal conjugate vaccines (PCVs) substantially reduced antibiotic consumption and contributed to an 84% decline in multidrug-resistant invasive pneumococcal disease following the introduction of PCV7 in the United States [80]. Influenza vaccinations have also been associated with reductions in inappropriate antibiotic prescribing for viral respiratory infections, lowering selection pressure for resistant strains [80].

In HTA, AMR-related benefits may be incorporated using extended CEA or cost-avoidance modeling that captures reductions in antibiotic consumption and emerging resistance [80,81]. Typical data sources include AMR surveillance systems, prescription databases, and studies linking reductions in infections to antibiotic use and resistance outcomes. Standardization of vaccination surveillance databases is urgently needed to allow better linkage with prescription databases and data on healthcare resource use [6].

### 3.18. Health System Strengthening

Strengthening of health systems is introduced as a new value element in this framework, capturing system-level benefits of vaccinations not explicitly reflected in the original ISPOR framework [45,82]. Although it overlaps with elements such as cost, productivity, and reductions in uncertainty, vaccination can contribute directly to system resilience, supporting its inclusion as a distinct domain.

Vaccination programs can strengthen health systems by improving surveillance capacity, cold-chain logistics, workforce training, and emergency preparedness. Evidence from national immunization programs, such as Vietnam’s Expanded Program on Immunization, shows that introduction of vaccines can stimulate investments that strengthen broader health infrastructure [41]. Preventing severe infections also helps maintain hospital and ICU capacity, supporting continuity of essential care during periods of high demand, as demonstrated by high-dose influenza vaccinations, which have been proven to reduce seasonal pressure on hospitals [82].

HTAs can draw on capacity-based modeling using indicators such as hospital and ICU utilization, cold-chain performance, and workforce deployment metrics [83,84]. Because vaccination often produces long-term system benefits, evaluations may need to consider longer time horizons rather than focusing only on short-term effects.

### 3.19. Real-Option Value

In the original ISPOR value framework, real-option value reflects the benefit of extending life so that patients may access more effective technologies in the future [19]. In this framework, the concept captures a forward-looking perspective relevant in rapidly evolving therapeutic areas where anticipated innovations may influence present decisions [85].

Unlike conventional CEA, which assumes a fixed treatment landscape, real-option value recognizes that survival gained today may enable patients to benefit from innovations that are not yet available [62,85]. While this may increase projected QALYs, it may also increase long-term costs, as illustrated in applications such as ipilimumab for metastatic melanoma [86]. For vaccines, real-option value can arise when early investment in platform technologies, such as mRNA manufacturing capacity or advanced procurement, allows healthcare providers to respond rapidly to future variants or emerging pathogens, even when the immediate benefits of vaccination are uncertain [9,87]. Thus, real-option value strengthens vaccine HTAs by highlighting preparedness, technological spillovers, and future pandemic response capacity.

In HTAs, real-option value can be operationalized using scenario modeling or real-options approaches that incorporate extended survival trajectories and future technology pipeline scenarios [88]. Typical data sources include survival curves from clinical trials, forecasts of therapeutic innovation, and expert-elicited probability estimates used to populate dynamic models of future health benefits.

### 3.20. Equity in Healthcare

In the original ISPOR value framework, “equity” reflects the importance of considering a broad distribution of health benefits [19]. In this framework, equity refers to reducing unfair differences in health outcomes and access to healthcare between populations, particularly for socially disadvantaged or high-risk groups [89].

Although widely recognized, practical integration of equity in HTAs remains limited. Equity can be incorporated through distributional CEA, which evaluates how health gains and opportunity costs are distributed across population subgroups [90]. For vaccines, equity is particularly relevant because immunization programs are often delivered at the population scale, reducing financial barriers and improving access for disadvantaged populations [91]. Vaccination may, therefore, contribute to narrowing health inequalities and strengthening the societal value of immunization programs. However, incorporating equity considerations may involve trade-offs with efficiency-based objectives, and careful modeling is required to avoid potential overlap with other value elements.

Common data sources include stratified health outcomes and coverage data based on factors such as income, geography, or ethnicity. Equity weights derived from stated-preference studies may also be used to adjust value assessments.

### 3.21. Scientific Spillover

In the original ISPOR value framework, “scientific spillovers” represent benefits that extend beyond the direct effects of a technology, including advances in knowledge, platform innovation, and future research productivity [19]. In this framework, the element is particularly relevant for vaccines because research and development (R&D) investments frequently generate transferable technologies.

The rapid development of COVID-19 vaccines using mRNA platforms illustrates how vaccine innovation can accelerate capabilities for other pathogens and therapeutic areas [92]. Similar spillover effects are observed in gene and cell therapies, where advances expand treatment possibilities beyond initial indications [93]. For HTAs, such spillovers are increasingly important as they may influence long-term cost-effectiveness and future healthcare delivery by enabling faster and more adaptive responses to emerging diseases [94]. Platform-based vaccine development (e.g., mRNA or viral vectors), therefore, strengthens pandemic preparedness and improves responsiveness to emerging pathogens [9,92].

Scientific spillovers are not yet routinely incorporated in HTAs but may be reflected through innovation metrics, expert elicitation, or modeling of research productivity. Typical indicators include patent counts, citation networks, R&D investment trends, and technology pipeline monitoring [93]. These indicators serve as proxies for knowledge diffusion and technological progress, translating into long-term societal value through accelerated innovation, improved preparedness for future outbreaks, and reduced development costs for subsequent vaccines.

## 4. Discussion

This study presents an adaptation of the original ISPOR value framework tailored to the specific characteristics of vaccination. While the original framework identified 12 potential sources of value [19], our expanded framework operationalizes 21 value elements to support a more comprehensive assessment of vaccines [95]. Core elements such as QoL and cost-effectiveness were retained and refined, while others were expanded to capture broader societal impacts (e.g., redefining productivity to include education and leisure and broadening fear of contagion to peace of mind). Additional elements particularly relevant to communicable disease prevention were introduced, including environmental impact, AMR control, health system strengthening, and real-world evidence as a standalone driver of value rather than simply a data source [95]. By mapping these 21 elements to HTA metrics, the framework aims to bridge conceptual theory and practical evaluation.

The COVID-19 vaccination rollout provides a relevant policy example of how broader vaccine value elements can influence real-world decision-making beyond traditional measures of efficacy and cost-effectiveness [7,19]. Vaccine policies such as rapid procurement, priority access for vulnerable groups, and strategic communication were influenced not only by clinical evidence but also by considerations including peace of mind, equity, macroeconomic recovery, and health system resilience. Equity-oriented allocation strategies prioritizing high-risk or underserved populations reflected social fairness objectives alongside clinical risk reduction [90,91]. Advance purchase agreements and investments in platform technologies demonstrated real-option value by preserving flexibility to respond to emerging variants [19,87]. Public communication emphasized reassurance and collective protection, highlighting the role of peace of mind [67]. Vaccination also supported economic reopening and labor market stability [73], contributed to scientific spillovers through rapid mRNA innovation [92]; vaccinations may have also reduced AMR by decreasing inappropriate antibiotic use [80]. Several of these benefits were central to the pandemic response yet remained outside the scope of traditional HTA frameworks [95].

As such, the experience of COVID-19 vaccination further validates this expanded framework. Decisions such as rapid procurement, equity-focused allocation, investments in platform technologies, and communication strategies aimed at public reassurance reflected value elements including peace of mind, system resilience, equity, and real-option value, even though these were not formally quantified in HTA processes [19,45,67]. These findings show that these broader elements are not only theoretically relevant but also influential in real-world policymaking. While this discussion does not constitute a formal empirical case study, it illustrates how expanded value frameworks may help structure broader considerations that were already influencing vaccine policy decisions during the pandemic. Incorporating a wider set of value dimensions can support more transparent prioritization in HTAs by linking economic, societal, system-level, and ethical considerations to established evaluation tools and stakeholder engagement processes [96]. Applying the revised, vaccine-adapted value framework retrospectively suggests that clinical evidence alone may not fully explain many pivotal policy decisions [45]. Future HTAs should, therefore, consider integrating these broader elements prospectively to better guide vaccine investment and strategies for preparedness.

The results demonstrate how the ISPOR value framework can be operationalized for vaccine HTAs by linking each value element to analytical tools familiar to values, such as QALYs, cost–benefit analysis, distributional CEA, VOI analysis, and macroeconomic or environmental assessments. This can help address critiques that the framework is conceptually rich but lacks practical guidance for implementation [19]. Future work could extend this framework to other health technologies beyond vaccines, such as orphan drugs or gene therapies.

Nonetheless, challenges remain. Some value elements, such as the value of hope and scientific spillover, remain difficult to quantify and may require proxy metrics or narrative justification [19,92]. Their adoption in LMICs may also require phased integration due to data limitations [97]. Assessing the value of vaccination often involves trade-offs across dimensions, reflecting the complexity of healthcare decision-making. Balancing cost and quality [45], individual and population health [98], equity and efficiency [45], and strategic resource allocation requires context-specific deliberation [45]. Similarly, promoting high adherence may conflict with cost-effectiveness goals [99], while reliance on RWE versus controlled trials introduces tensions between generalizability and internal validity [99].

Improvements in QoL must also be weighed against budgetary constraints [38], and environmental impacts should not compromise health outcomes [100]. In some cases, patient preferences, such as route of administration, may conflict with broader public health objectives, including coverage or equity [80]. Societal gains, such as the reduced burden of informal care and increased leisure time, must also be balanced against economic productivity impacts [47]. Navigating these trade-offs requires HTAs to adopt a broader, integrated perspective so that vaccination policies align with societal priorities and long-term public health goals. Some value elements may also appear conceptually related; examples include productivity, macroeconomic impact, and informal care, uncertainty, and real-world evidence. To avoid double-counting, these elements should be assessed within clearly defined analytical boundaries rather than aggregated indiscriminately. In practice, analysts may select relevant elements depending on the evaluation perspective (e.g., healthcare system or societal) and ensure overlapping effects are not counted more than once. This framework, therefore, provides a structured catalog of value dimensions rather than implying that all elements must be simultaneously quantified in a single HTA.

Clinical efficacy and safety are fundamental determinants of vaccine value and are included as a separate domain in this framework. They strongly influence downstream outcomes such as health gains and cost-effectiveness. In some HTA decisions, improved safety without loss of efficacy becomes decisive. Vaccines provide a clear example: the transition from whole-cell to acellular pertussis vaccines was largely driven by a more favorable safety profile despite comparable effectiveness [101]. RWE is included because it plays a key role in demonstrating and refining vaccine value in real-world settings, particularly by improving estimates of long-term effectiveness, indirect protection, and population-level impacts that may not be fully captured in clinical trials.

### 4.1. Policy and Implementation Considerations

The proposed framework should not be interpreted as requiring all 21 value elements to be quantified in every HTA. Rather, it offers a structured set of value dimensions that can be selected according to the context of a country, data availability, and decision needs. In practice, HTA bodies can apply this framework stepwise by identifying relevant elements, choosing suitable methods (e.g., CEA, distributional analysis, or VOI), and integrating the results into policy deliberation. HICs may gradually incorporate broader societal and system-level elements, and LMICs may begin with a smaller set of measurable dimensions and expand over time as data systems and methodological capacity improve.

In addition, the feasibility of incorporating broader value elements should be considered in relation to C-E objectives. In resource-constrained settings, particularly in LMICs and LICs, the inclusion of additional value dimensions may increase analytical complexity and data requirements and therefore should be balanced against available resources and decision-making needs.

Challenges will still be encountered during implementation, including limited data, difficulty measuring some elements such as scientific spillovers or peace of mind, and methodological complexity in incorporating broader societal effects. Feasibility also depends on institutional capacity, stakeholder engagement, and coordination between HTA and public health bodies. Transparent reporting, sensitivity analyses, and gradual methodological development may help address these barriers while ensuring consistency in vaccine evaluation.

### 4.2. Limitations

This study has several limitations. First, the framework was developed through a targeted scoping search rather than a full systematic review, so some relevant publications may have been missed. Second, the findings remain conceptual and have not yet been empirically validated in real-world HTA applications. Third, several value elements may be difficult to quantify, especially in resource-constrained settings, due to data and methodological limitations. Broader value dimensions may also be harder to integrate into traditional cost-effectiveness-based frameworks. In addition, the incorporation of broader value elements may pose challenges for traditional cost-effectiveness frameworks, particularly in LMICs and LICs, where resource constraints and data limitations may restrict the feasibility of such approaches. Finally, the framework was developed through a synthesis of the literature and the author’s interpretation rather than a formal consensus process. Future research should, therefore, validate and refine these findings through structured stakeholder engagement.

## 5. Conclusions

This study provides a structured, conceptually grounded revision of the ISPOR value framework tailored to vaccines, offering pathways to operationalize broader value elements within HTA. By addressing longstanding critiques around practicality, prioritization, and trade-offs, this study may support more coherent and transparent evaluation. As healthcare systems worldwide navigate post-pandemic recovery and intensifying demands on preventive care, this framework may help inform more equitable, sustainable, and accountable vaccine decision-making across both HIC and LMIC settings.

## Figures and Tables

**Table 1:** Illustrative mapping of value flower elements identified by HTA tools and data sources.

Value Element (Petal)	HTA Metric/Evaluation Tool	Typical Data Sources
Vaccine Efficacy and Safety	QALYs, DALYs, and CEA	RCTs and Phase III data
Real-World Evidence/Data	Adjusted CEA, risk modeling	Registries and EHRs
Quality of Life	Utility values in QALYs	EQ-5D, SF-36, and PROs
Cost, Savings, and Budget Impact	BIA and ICER	Claims data and cost studies
Informal Care	Indirect cost estimation	Time-use surveys and caregiver interviews
Productivity and Leisure	Human capital and friction cost	Labor stats and economic studies
Disease Severity	Severity-weighted QALYs	Burden of disease reports
Adherence, Compliance, and Persistence	Scenario sensitivity in models	Coverage data
Insurance Value	Willingness to pay (WTP)-based valuations; extended CEA	Stated preference surveys and health and household surveys
Peace of Mind	Contingent valuation	Survey research and qualitative data
Patient Preferences	Discrete choice experiments	Preference elicitation studies
Value of Hope	Risk-weighted utility and preference-sensitive modeling	Psychological research and case studies
Macroeconomic Impact	CGE models	GDP stats and labor models
Environmental Impact	Environmental life-cycle assessment	Supply chain audits and emission databases
Controlling AMR	Cost-avoidance modeling	Resistance rates and antibiotic use
Health System Strengthening	Capacity-based value assessment	ICU use and infrastructure metrics
Real-Option Value	Real-options analysis and scenario modeling	Survival projections and epidemiological forecasts
Equity	Distributional CEA	Stratified outcome data
Scientific Spillover	Innovation indicators and knowledge diffusion metrics	Patent counts, citation networks and R&D investment data
(Un)certainty in Information	Probabilistic sensitivity analysis and VOI analysis	Parameter distributions from the literature, RWE, epidemiological models, and expert elicitation
Cost-Effectiveness	ICER, CEA, and CUA	Aggregated from above

Abbreviations used in this table: AMR = Antimicrobial Resistance; BIA = Budget Impact Analysis; CEA = Cost-Effectiveness Analysis; CGE = Computable General Equilibrium; CUA = Cost-Utility Analysis; DALYs = Disability-Adjusted Life Years; EHRs = Electronic Health Records; EQ-5D = EuroQol 5-Dimension health questionnaire; HTA = Health Technology Assessment; ICER = Incremental Cost-Effectiveness Ratio; PROs = Patient-Reported Outcomes; QALYs = Quality-Adjusted Life Years; RCTs = Randomized Controlled Trials; SF-36 = Short-Form 36-Item Health Survey; WTP = Willingness-To-Pay; GDP= Gross Domestic product; VOI= Value of Information.

## Data Availability

No new data were created or analyzed in this study. Data sharing is not applicable to this article.

## Supplementary Materials

The following supporting information can be downloaded at: https://www.mdpi.com/article/10.3390/jmahp14020024/s1, File S1: Search strategies used for the targeted rapid scoping search in PubMed and Scopus.

## Abbreviations

The following abbreviations are used in this manuscript:

AMR	Antimicrobial Resistance
BIA	Budget Impact Analysis
CEA	Cost-Effectiveness Analysis
CGE	Computable General Equilibrium
CUA	Cost-Utility Analysis
DALYs	Disability-Adjusted Life Years
EHRs	Electronic Health Records
EQ-5D	EuroQol 5-Dimension
HICs	High-Income Countries
HTA	Health Technology-Assessment
ICER	Incremental Cost-Effectiveness Ratio
ISPOR	International Society for Pharmacoeconomics and Outcomes Research
LMICs	Low- and Middle-Income Countries
QALYs	Quality-Adjusted Life Years
QoL	Quality of Life
RCTs	Randomized Controlled Trials
RWD/RWE	Real-World Data/Real-World Evidence
WTP	Willingness-To-Pay
