# Supplementary material for: Introducing an Expanded Value Framework in Health Technology Assessment of Vaccines
Source: J Mark Access Health Policy. 2026 Apr 28;14(2):24. doi: 10.3390/jmahp14020024 (PMC13214781; doi:10.3390/jmahp14020024)
Supplement: Supplementary file 1 [file jmahp-14-00024-s001.zip › supplemenatry file.pdf]

Supplementary S1 – Search strategy

Database: PubMed
Search string:
((("vaccine"[Title/Abstract] OR "vaccination"[Title/Abstract] OR "immunization"[Title/Abstract] OR "immunisation"[Title/Abstract])
AND
("value framework"[Title/Abstract] OR "value frameworks"[Title/Abstract] OR "value element*"[Title/Abstract] OR "broader value"[Title/Abstract] OR "societal value"[Title/Abstract] OR "economic value"[Title/Abstract])
AND
("health technology assessment"[Title/Abstract] OR "HTA"[Title/Abstract] OR "economic evaluation"[Title/Abstract] OR "cost-effectiveness"[Title/Abstract] OR "cost benefit"[Title/Abstract] OR "cost-benefit"[Title/Abstract] OR "cost utility"[Title/Abstract] OR "cost-utility"[Title/Abstract]))
AND ("2010/01/01"[Date - Publication] : "2025/12/31"[Date - Publication])

Database: Scopus
Search string:
TITLE-ABS(
(vaccine OR vaccination OR immunization OR immunisation)
AND
("value framework" OR "value frameworks" OR "value element*" OR "broader value" OR "societal value" OR "economic value")
AND
("health technology assessment" OR HTA OR "economic evaluation" OR "cost-effectiveness" OR "cost benefit" OR "cost-benefit" OR "cost utility" OR "cost-utility")
)
AND PUBYEAR > 2009
AND PUBYEAR < 2026
